# Anti-proliferative effects of mesenchymal stem cells (MSCs) derived from multiple sources on ovarian cancer cell lines: an in-vitro experimental study

**DOI:** 10.1186/s13048-019-0546-9

**Published:** 2019-07-27

**Authors:** C. Khalil, M. Moussa, A. Azar, J. Tawk, J. Habbouche, R. Salameh, A. Ibrahim, N Alaaeddine

**Affiliations:** 1 Regenerative Medicine and Inflammation Laboratory, Faculty of Medicine, Saint-Joseph University, Beirut, Lebanon; 2 Reviva Research and Application Center-Lebanese University, Middle East Institute of Health University Hospital, Beirut, Lebanon; 3 Faculty of Medicine, Lebanese University, Beirut, Lebanon; 4 Neuroscience Research Center, Faculty of Medical Sciences, Lebanese University, Beirut, Lebanon

**Keywords:** Mesenchymal stem cells, Adipose tissue, Bone marrow, Umbilical cord, Ovarian cancer cell lines

## Abstract

Mesenchymal stem cells (MSCs) have surfaced as ideal candidates for treatment of different therapeutically challenging diseases however their effect on cancer cells is not well determined. In this study, we investigated the effect of MSCs derived from human bone marrow (BM), adipose tissue (AT), and umbilical cord derived MSCs (UC-MSCs) on ovarian cancer.

Measurements of ovarian tumor marker proteins were computed by ELISA. Proliferative, apoptosis and anti-inflammatory effects of the MSCs were measured by Flow cytometry (FCM). MMPs expression was measured by RT-PCR.

The co-culture of cancer cell lines OVCAR3, CAOV3, IGROV3 and SKOV3 with the conditioned media of MSCs (CM-MSC) and MSCs showed an increase in cellular apoptosis, along with a reduction in the level of CA-125 and a decline of LDH and beta-hCG. A decrease in CD24 of the cancer cell lines in co-culture with the CM-MSCs showed a reduction of the cancer tumorigenicity. In addition, the invasion and aggressiveness of cancer cell lines was significantly decreased by CM-MSC; this was translated by a decrease in MMP-2, MMP-9, and CA-125 mRNA expression, and an increase in TIMP 1, 2, and 3 mRNA expression. An increase in IL-4 and IL-10 cytokines, and a decrease in GM-CSF, IL-6, and IL-9, were also noted.

In conclusion, mesenchymal stem cells derived from different sources and their conditioned media appear to have a major role in inhibition of cancer aggressiveness and might be considered as a potential therapeutic tool in ovarian cancer.

## Introduction

Ovarian cancer is the lethal of all gynecologic malignancies. Expected projections in 2018 estimate approximately 22,240 new diagnoses and 14,070 female deaths in the United States alone [1]. Mortality in ovarian cancer is predominantly due to tumor recurrence and acquired chemoresistance [2–4]. Tumor recurrence is common because majority of people are diagnosed at advanced stages of the disease [5, 6].

Historically, ovarian carcinosarcomas were often treated with the same first-line chemotherapy as uterine carcinosarcomas, ifosfamide/paclitaxel [7–11]. Multiple phase II studies have also demonstrated efficacy with the use of carboplatin/paclitaxel in uterine carcinosarcomas [8, 12, 13]. Additionally, several single institution studies have provided convincing evidence that ovarian carcinosarcoma patients have lower response rates to chemotherapy and poor overall and disease-specific survival [14, 15]. Thus, the preferred first-line treatment for ovarian carcinosarcoma patients remains debatable [16–18].

Mesenchymal stem cells (MSCs) have emerged as ideal agents for the restoration of damaged tissues in clinical applications due to their undifferentiated cell characterization, self-renewal ability with a high proliferative capacity, their paracrine, trophic effect and their mesodermal differentiation potential [19, 20]. Additionally, they produce bioactive anti-inflammatory agents and support regeneration of injured tissues [19, 21, 22].

The therapeutic potential of MSCs has been explored in a number of phase I, II, and III clinical trials [23], of which several were targeted against graft versus-host disease and to support engraftment of hematopoietic stem cells [24, 25]. Yet, very few of these trials use MSCs to treat tumor diseases [23, 26, 27], such as gastrointestinal, lung, and ovarian cancer. As for the study targeting ovarian cancer, MSCs expressing cytokines were used as therapeutic payload [23] and were derived from bone marrow.

Bone marrow (BM) was the first source of MSCs reported as a potential candidate for cell replacement therapy [28] followed by adipose tissue (AT) [29] and more recently umbilical cord (UC) [30, 31]. Compared to BM, AT and UC are favorably obtained by less invasive methods. AT contains similar stem cells termed as processed lipoaspirate (PLA) cells, which can be isolated from cosmetic liposuctions in large quantities and grown easily under standard tissue culture conditions [26]. MSCs derived from UCs have the potential to be cultured longest and have the highest proliferation capacity compared to the other sources [26].

Effective curative therapy for ovarian cancer has yet to be developed according to the current status of MSC-based therapeutic approaches for cancer. This study seeks to investigate the potential of mesenchymal stem cells derived from AT, BM, and UC as promising sources of anti-tumor effects on ovarian cancer.

## Materials and methods

### Collection of MSCs

Ten collections were made from each MSC source (BM, UC, and AT). BM aspirates were obtained by puncturing the iliac crest of participants ranging in age from 30 to 60 years at the hematology department of the Middle East Institute of Health University Hospital. UC units were collected from the unborn placenta of full-term deliveries in a multiple bag system containing 17 mL of citrate phosphate dextrose buffer (Cord Blood Collection System; Eltest, Bonn, Germany) [26] and processed within 24 h of collection. AT was obtained from participants ranging in age from 26 to 57 years, who underwent liposuction at Hotel Dieu de France Hospital (HDF) (Beirut, Lebanon). The Saint-Joseph University and the HDF Ethics Review Board approved the retrieval of all MSC collections (approval reference number: CEHDF1142), and all patients were asked to read and approve/sign informed consent forms prior to any participation.

### Isolation and culture of mononuclear cells from BM

The aspirates were diluted 1:5 with 2 mM ethylenediaminetetraacetic acid (EDTA)-phosphate-buffered saline (PBS) (Sigma-Aldrich). The mononuclear (MNC) fraction was isolated by density gradient centrifugation at 435 *g* for 30 min at room temperature using Ficoll-Hypaque-Plus solution (GE Healthcare BioSciences Corp) and seeded at a density of 1 × 10^6^ cells per cm^2^ into T75 or T175 cell culture flasks (Sigma, Aldrich). Within 3 days after isolation, the first change of medium was accomplished. The resulting fibroblastoid adherent cells were termed BM-derived fibroblastoid adherent cells (BM-MSCs) and were cultivated at 37 °C at a humidified atmosphere containing 5% CO_2_. The expansion medium consisted of Dulbecco’s modified Eagle’s medium-alpha modification (Alpha-MEM) + 10% fetal bovine serum (FBS; Invitrogen; Thermo Fisher Scientific, Inc., Waltham, MA, USA) and 5% penicillin-streptomycin-amphotericin B solution (PSA: Hyclone; GE healthcare, Logan, UT, USA). BM-MSCs were maintained in Alpha-MEM + 10% FBS and 5% PSA until they reached 70 to 90% confluency. Cells were harvested at subconfluence using Trypsin (Sigma-Aldrich). Cells at the second passage and thereafter were replated at a mean density of 1.3 ± 0.7 × 10^3^/cm^2^.

### Isolation and culture of MSC from human umbilical cord Wharton jelly

Umbilical cord was collected in PBS supplemented with 10% PSA and transferred to the laboratory in a maximum of 12 h. After washing, cord samples were cut into 1-2 cm sections, the umbilical vessels were removed (artery and veins), and Wharton’s jelly was collected and minced into pieces, then digested by collagenase overnight and then cultured in flasks.

Nonadherent cells were removed 12 h after initial plating. The same culture conditions and media were applied as described for BM-MSCs. Adherent fibroblastoid cells only (UCMSC) appeared as CFU-F and were harvested at subconfluence using Trypsin (Sigma-Aldrich). Cells at the second passage and thereafter were replated at a mean density of 3.5 ± 4.8 × 10^3^/cm^2^.

### Isolation and culture of PLA cells from AT

To isolate the stromal vascular fraction (SVF), lipoaspirates were washed intensely with PBS containing 5% of PSA. Next, the lipoaspirates were digested with an equal volume of 0.075% collagenase type I (Sigma-Aldrich) for 30–60 min at 37 °C with gentle agitation. The activity of the collagenase was neutralized with DMEM containing 10% fetal bovine serum (FBS; Invitrogen; Thermo Fisher Scientific, Inc., Waltham, MA, USA). To obtain the high-density SVF pellet, the digested lipoaspirate was centrifuged at 1,200 *g* for 10 min. The pellet was then resuspended in DMEM containing 10% FBS and filtered through a 100 μm nylon cell strainer (Falcon). The filtered cells were centrifuged at 1,200 *g* for 10 min. The resuspended SVF cells were plated at a density of 1 × 10^6^/cm^2^ into T75 or T175 culture flasks. Nonadherent cells were removed 12–18 h after initial plating by intensely washing the plates. The resulting fibroblastoid adherent cells, termed AT-derived fibroblastoid adherent cells (ADMSC), were cultivated under the same conditions as described for BM-MSCs. ADMSCs were harvested at subconfluence using Trypsin (Sigma-Aldrich). Cells at the second passage and thereafter were replated at a mean density of 1.8 ± 3.1 × 10^3^/cm^2^.

### Co-culture of MSCs with cancer cell lines

Human ovarian epithelial cancer cell lines SKOV3, OVCAR3, IGROV3, and CAOV3 were purchased from the American Type Culture Collection (ATCC; Manassas, VA, USA) and cultured in DMEM/F12 supplemented with 10% FBS supplemented with 5% PSA. The cells were cultured at 37 °C in a humidified atmosphere with 5% CO_2_.

### Conditioned media (CM) preparation

ADMSC, BM-MSC, and UCMSC were cultured with DMEM/F12 supplemented with 5% PSA at subconfluency. Thereafter, the supernatant, containing all released cytokines and chemokines to be studied, was collected.

### Co-culture maintenance

Only ADMSC, BM-MSC, and UCMSC before passage 3 were used for co-culture. OVCAR3, SKOV3, IGROV3, and CAOV3 were cultured in direct contact with ADMSC, BM-MSC, and UCMSC cells in DMEM/F12 with 5% PSA and in a sterile humidified incubator with 5% CO_2_ at 37 °C for 48 h, and with the supernatant under the same conditions. The co-culture ratio of ADMSCs, BM-MSCs, and UCMSCs to SKOV3, OVCAR3, IGROV3, and CAOV3 cells was 1:1. Cell lines were considered the control group and underwent the same culturing time and conditions.

### Assessing differentiation potential of MSCs

The cultured cells were differentiated into osteogenic, adipogenic and chondrogenic lineage by culturing in osteogenic medium [DMEM supplemented with 10^− 8^ M dexamethasone (Sigma, D4902), 10 mM β glycerophosphate (Sigma, G9422), and 50 μg/ml ascorbic acid], adipogenic medium [DMEM supplemented with 10 mM 3 isobutyl-1-methylxanthine (Sigma, 17018), 0.1 mM indomethacin (Sigma, 17378), 10 μg/ml insulin (Sigma, I6634), 10^− 6^ dexamethasone] and chondrogenic medium (Stempro, Invitrogen) and confirmed by staining with Alizarin red (Sigma, A5533), Oil red O (Sigma, O0625) and Alcian blue (Himedia Laboratories, Mumbai, India: Cat no RM471-1 g staining, respectively).

### Flow cytometry analysis

For surface marker immunophenotyping, cells were stained with the following conjugated antibodies: anti-CD45-vioblue, anti-CD34-PE, HLADr-vioblue, anti-CD73-PE, anti-CD90-FITC, anti-CD105-vioblue, CD24-PE CD44-FITC, CD133-APC, CD14-PE and relevant isotypes (Miltenyi-Biotec). We acquired at least 20,000 events as test samples.

### Cytokine analysis

For the cytokine analysis, we used the MACSplex Cytokin12 kit. Supernatants were mixed to capture specific beads for each cytokine: granulocyte/macrophage colony-stimulating factor (GM-CSF), interferon (IFN)-α, IFN-γ, interleukin (IL)-2, IL-4, IL-5, IL-6, IL-9, IL-10, IL-12p70, IL-17A, and tumor necrosis factor (TNF)-α. Antibodies conjugated with PE were added and incubated for 2 h at room temperature and away from light. After centrifugation, the bead-containing pellets were resuspended. The MACSQuant® express mode was used to perform flow cytometric acquisition and data analysis. Background signals were resolved by analyzing beads only incubated with the cell culture medium. The background signals were subtracted from the bead signals incubated with supernatants.

### RNA extraction and quantitative real-time RT-PCR

Total RNA was extracted from samples using QIAamp RNA extraction Kit (Qiagen Inc., Valencia, CA, USA). RNA quality and yields were analyzed using nanodrop. Complementary DNA (cDNA) was synthesized from 500 ng of total RNA in a 20 μL reaction solution using iScript™ Cdna-synthesis Kit (Bio-Rad Laboratories, CA).

Quantitative real-time PCR was performed with the iQ™ SYBR® Green Supermix (Bio-Rad Laboratories, CA) in triplicate. The reaction conditions were: polymerase activation at 95 °C for 5 min, 40 cycles of denaturation at 95 °C for 20 s, and annealing and extension at 62 °C for 20 s. The relative quantification of gene expression was normalized to the expression of endogenous GAPDH. Real-time PCR was performed with multiple https://blast.ncbi.nlm.nih.gov/blast.cgi sequences as indicated in Table 1.

**Table 1:** List of primer sequences for real time PCR

Primers	Forward	Reverse
CA-125	5′-GCACAATTCCCCCAACCTCC−3′	5′-TGCCTCTGGATGGAGGTCTAA−3′
MMP-2	5′- AGCTGGCCTAGTGATGATGTT −3′	5′- TTCAGCACAAACAGGTTGCAG−3′
MMP-9	5′-CGCAGACATCGTCATCCAGT−3′	5′-GAAATGGGCGTCTCCCTGAA−3′
TIMP-1	5′GACCAAGATGTATAAAGGGTTCCAA−3′	5′GAAGTATCCGCAGACACTCTCCAT−3′
TIMP-2	5′-AGGCGTTTTGCAATGCAGAT−3′	5′TCCAGAGTCCACTTCCTTCTCACT−3′
TIMP-3	5′-CAGGACGCCTTCTGCAACTC−3′	5′-AGCTTCTTCCCCACCACCTT−3′
GAPDH	5′-GCACCACCAACTGCTTAGCA−3′	5′-CTTCCACGATACCAAAGTTGTCAT−3′

### Apoptosis test

Apoptosis was evaluated by staining cell cultures with 10 μL Annexin V (fluorescein isothiocyanate: Miltenyi-Biotec Annexin V- FITC kit) at 4 °C for 20 min in the dark, followed by counterstaining with 5 μL propidium iodide (PI) at room temperature for 5 min in the dark. Detection by flow cytometry was analyzed using a MACSQuant analyzer device for each 10^4^ cell sample, and calculation of apoptosis cells was performed using MACSQuant computer software.

Examination of apoptosis was performed by 4, 6-diamidino-2-phenylindole dihydrochloride (DAPI) staining. Cell lines were cultured on glass slides for 48 h with ADMSC, BM-MSC and UCMSC or their supernatant, while the controls of cells were cultured in serum-free media. After a 48 h incubation period, the slides were rinsed with PBS and fixed in 4% formaldehyde for 10 min at room temperature. Following two washes with PBS, cells were stained with DAPI staining diluted in PBS for 15 min. The slides were then visualized on a fluorescence microscope.

### Tumor markers assay: CA-125, CA-19-9, AFP, CEA, LDH, and beta-hCG

CA-125, CA-19-9, AFP, CEA, LDH, and beta-hCG were measured by ELISA. The supernatant and the antibody (monoclonal biotinyle) form a complex after the interaction of biotin and streptavidin during incubation for 60 min. The excess was eliminated by washing and an enzyme-antibody complex is added. The complex antibody-enzyme formed the final sandwich complex. After incubation, the excess was washed again. Afterwards, sulfuric acid was added to stop the reaction and the solution’s color transformed from blue to yellow. The color intensity was directly correlated to the concentration of samples. The absorbance was read at 450 nm.

### Statistical analyses

Statistical significance was determined based on a paired *T*-test and one-way ANOVA test. *P* values below 0.05 were considered as statistically significant.

## Results

### Phenotyping and characterization of BM, UC, and AT derived MSCs

**Fig. 1:**
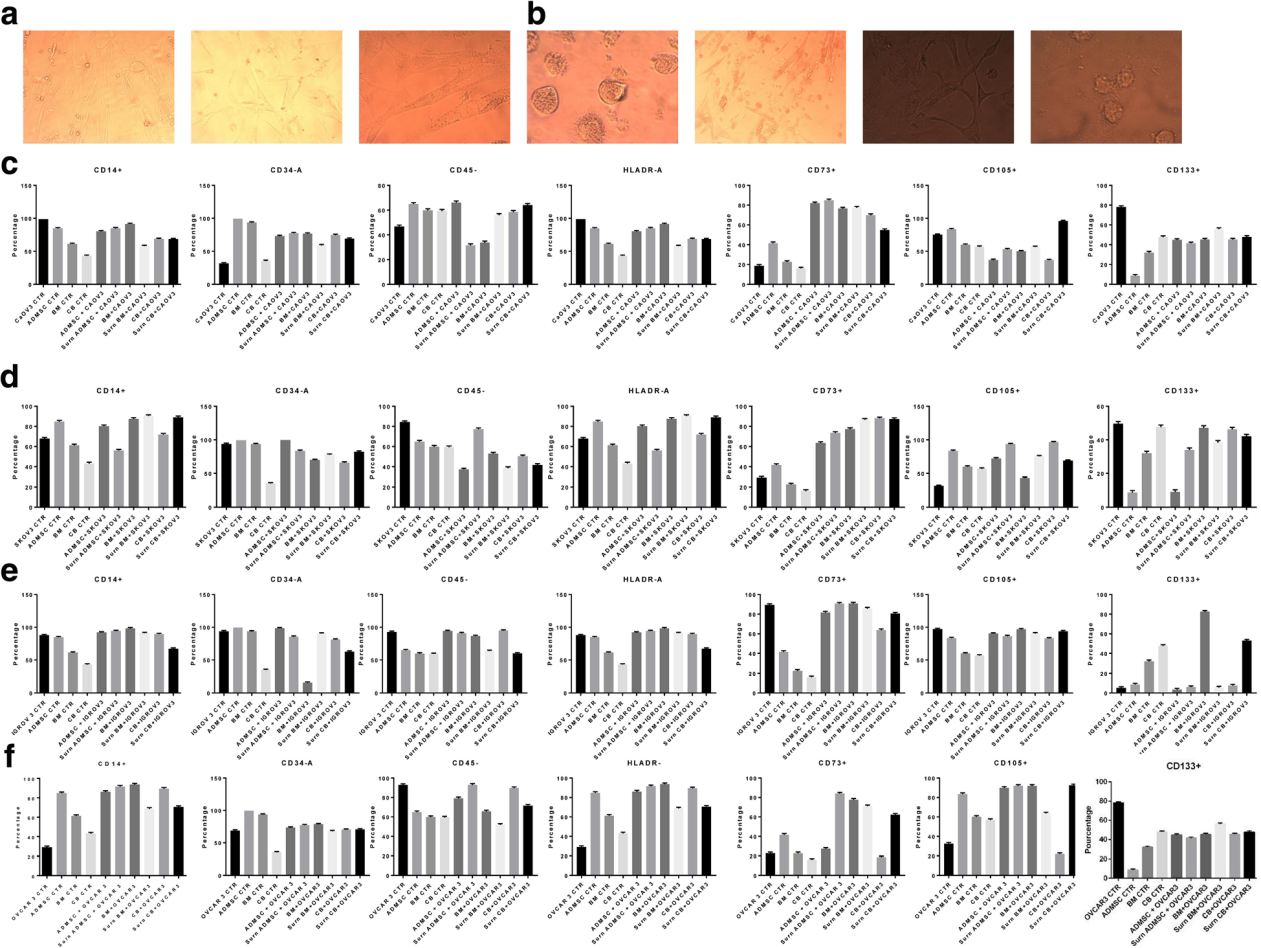
Morphology and flow cytometry analysis. **a-b** Morphology of MSC from AD, BM and CB. Scale bar, 100 μm. Morphological observation of OVCAR3, SKOV3, IGROV3 and CAOV3. Scale bar,100 μm. **c-f** Representative FCM result of MSC markers: CD34, CD45, HLADr, CD14, CD105, CD90, CD73, CD44, and CD24. All MSCs were positive for CD105, CD90, CD44, and CD73, and negative for the leukocyte common antigen CD45, the hematopoietic lineage marker CD34, the cancer marker CD24, and the macrophage markers HLADr and CD14 (Table 1). FCM results of CAOV3, OVCAR3, IGROV3 and SKOV3 in co-culture with MSCs and CM-MSCs. Error bars represent the means ± SD, *n* = 5; * *p* = 0.051, **p = 0.005, ***p < 0.001,****p < 0.0001

At an initial plating density of 1 × 10^6^ cells per cm^2^, both BM-and AT-derived MSCs formed a monolayer 4–5 days after initial plating. In contrast, UCMSCs were initially detected 2–4 weeks after plating when applying the same initial plating density. Regardless of the cell source, a fibroblastic cell like morphology was detected in all cell lines (Fig. 1a).

For further characterization of the MSCs, surface protein expressions were examined by flow cytometry. All MSCs, derived from the three different sources expressed CD44, CD73, CD105, and CD90 and were negative for CD14, CD34, CD45, HLADr, CD133, and CD24. CD105 was more expressed on BM- and ADMSCs than on UCMSCs (Fig. 1c, d, e, f).

### Differentiation potential of MSCs

**Fig. 2:**
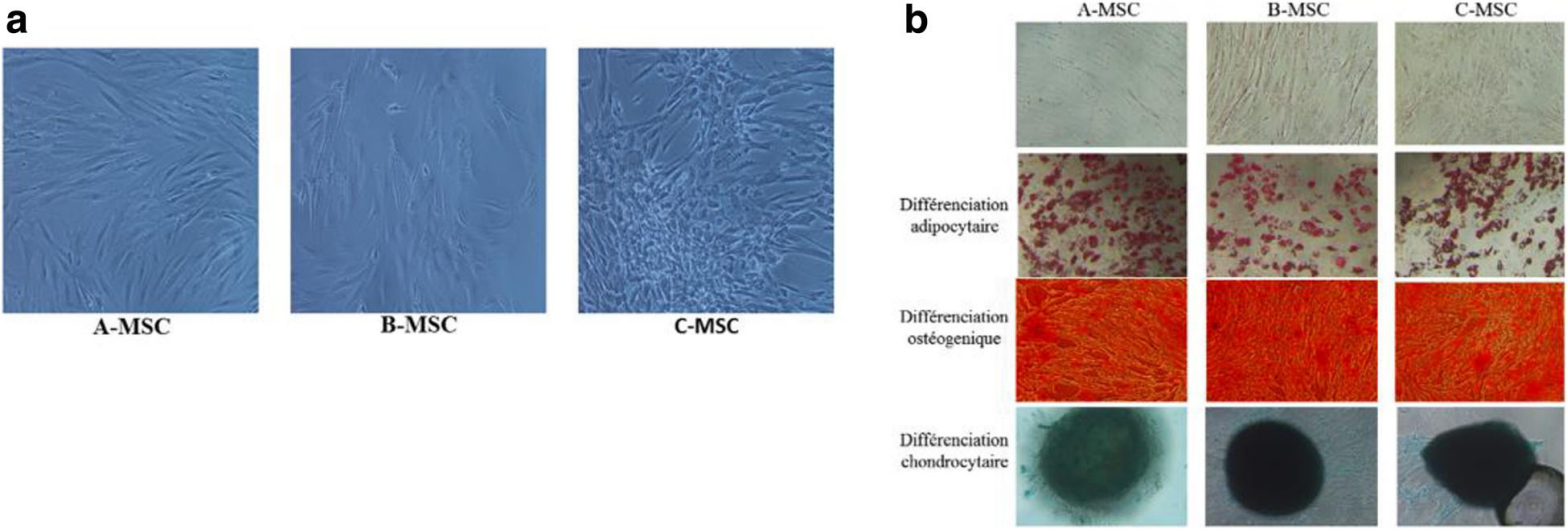
Differentiation capacity of MSCs. Image (**a**) Morphology of MSC from Different sources. Cells were incubated for 3 weeks with adipogenic, osteogenic, and chondrogenic media. Representative images (**b**) of differentiated cells first row represent day 0 of coloration. Second row shows that the intracellular lipid droplets were confirmed by Oil Red O staining compared to the control cells. The third row, the presence of calcium deposit was visualized by Alizarin Red staining compared to the control. The presence of GAG in the last row was confirmed with Alcian Blue staining and the solid chondrogenic micromass compared to the control

To further confirm the identity of our cell lines the cells were differentiated into chondrocytes, adipocytes and osteocytes as seen in the (Fig. 2a, b, c) where Oil O red indicate the presence of adipocytes, Alizarin red indicate osteocytes formation and Alcian blue indicate chondrocytes formation.

### Immunophenotyping and characterization of cancer cell lines

After isolation and plating the cancer cell lines OVCAR3 and IGROV3 showed a clustered morphology whereas SKOV3 and CAOV3 showed a fibroblastic cell morphology (Fig. 1b). Flow cytometry analysis shows that the above cancer cell lines express similar cell markers as our MSCs except the CD24 which is known to modulate growth and differentiation of cancer cells. OVCAR3, and CAOV3 were found to be positive for CD44, CD73, CD105, CD90, CD133 and CD24 and negative for CD14, CD34, CD45, HLADr. SKOV3 was positive CD44, CD73, CD105, CD90, CD133 and negative for CD14, CD34, CD45, HLADr and low for CD24. IGROV3 was positive for CD44, CD73, CD105, CD90, and CD24 and negative for CD133 CD14, CD34, CD45 and HLADr. (Fig. 1c, d, e, f).

### Inhibition of tumor markers by MSCs derived from AT, BM and UC

**Fig. 3:**
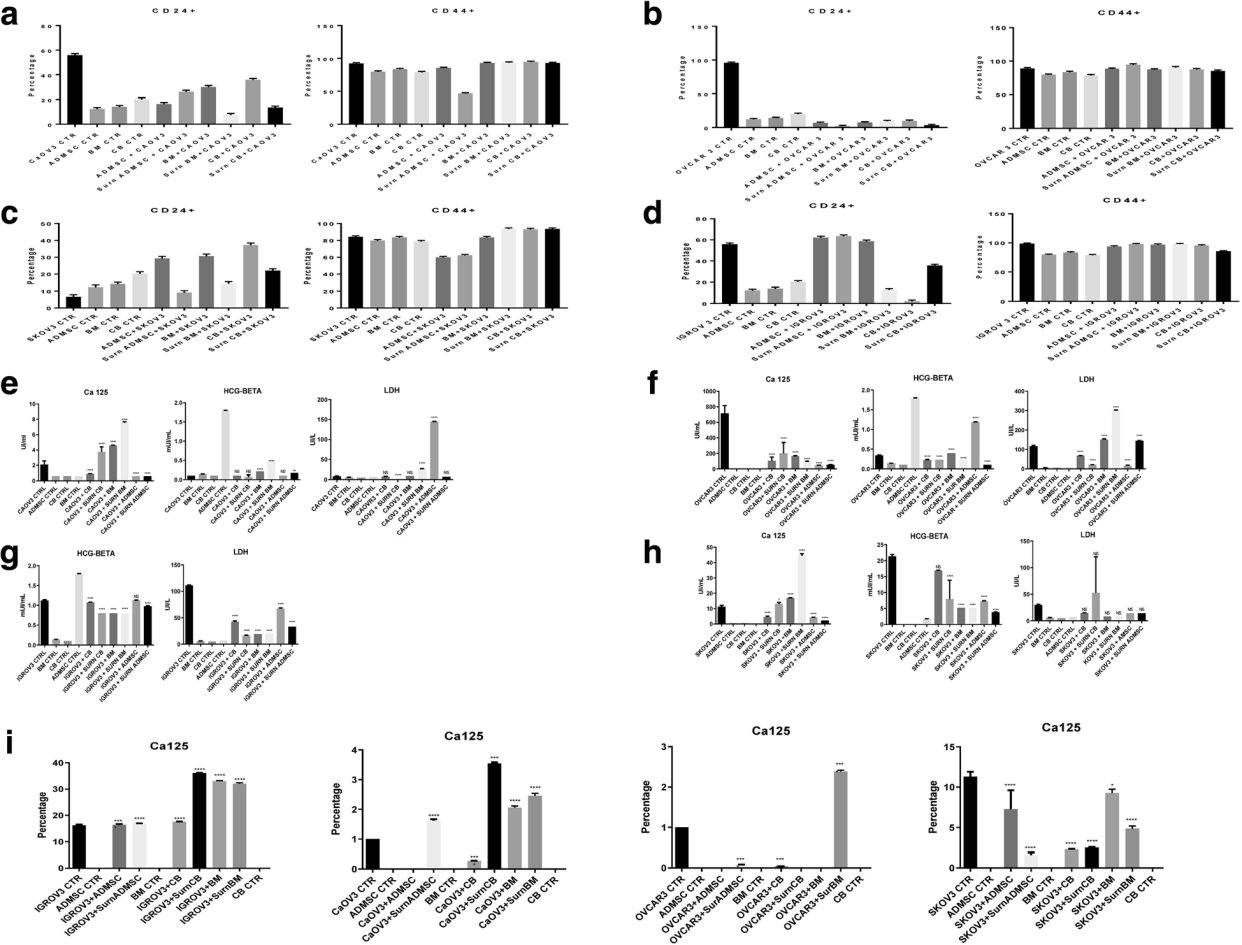
Expression of Tumor markers. **a-d** CD44+/CD24- expression in CAOV3, OVCAR3, IGROV3. **e-h** Cell culture supernatant from co-culture was collected, and undiluted samples were analyzed for detection of CA-125, LDH and beta-hCG. The concentration of secretion is shown in the x-axes. The limits of detection (IU/ml) were CA-125: 0.95, beta-HCG: 0.99, LDH = 0.99. **i** Real time PCR showed a decrease in Ca-125 levels in CAOV3, SKOV3 and OVCAR3, in mRNA levels. The results were displayed as percentage of controls. Error bars represent the means ± SD, n = 5; * p = 0.051, **p = 0.005, ***p < 0.001,****p < 0.0001

In order to study the effect of MSCs on Tumor Cell marker flow cytometry analysis were performed on CD24 and CD44. Our results showed a significant decrease in the level of CD24 when OVCAR3, CAOV3, IGROV3 and SKOV3 were co-cultured with BMMSC, UCMSC and ADMSC and CM-MSCs. (*p* < 0.0001) (Fig. 3a, b, c, d).

CA-125, CEA, AFP, LDH, CA-19-9, and beta-hCG are known to be expressed in ovarian cancer and used as tumor markers. We performed an ELISA test to detect their level when co-cultured with MSCs. A significant decrease in CA-125 expression in OVCAR3, SKOV3 and CAOV3 is seen when incubated with the supernatant of CB and AT (75–90%, *p* = 0.0017). CA-125 is not expressed in IGROV3. Also, a decrease in the secretion of LDH (10–20%, *p* = 0.0003) and beta-hCG (16–20%, *p* = 0.04) were observed in all cell lines. Similar results were found by co-culture of cell lines in direct contact with the MSCs, however it was more drastic with the CM-MSCs (Fig. 3e, f, g, h).

### Inhibition of cell proliferation and apoptosis

**Fig. 4:**
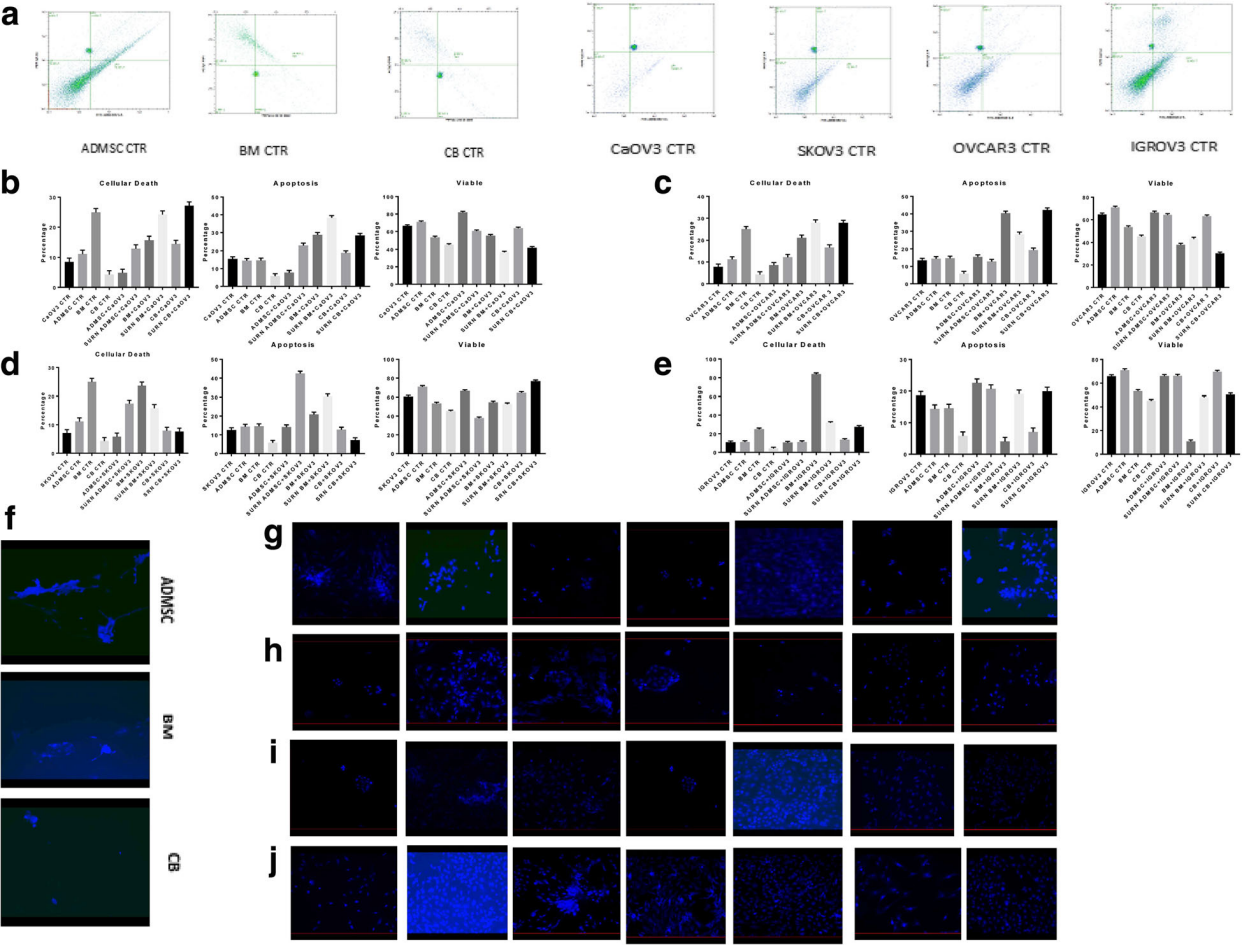
Apoptosis and cell death. **a-e** FCM using Annexin V /PI showed an increase in cell death in cancer cell lines. The increase rate was calculated by subtracting value from the control. Error bars represent the means ± SD, n = 5; * p = 0.051, **p = 0.005, ***p < 0.001,****p < 0.0001. **f-j** DAPI staining revealed an increase in blue fluorescence relative to the co-culture with MSC and/or supernatant. **f** MSCs control. First row (**g**): CAOV3 CTR, CAOV3 with ADMSC, CAOV3 with BM-MSC, CAOV3 with UC-MSC, CAOV3 with CM-ADMSC, CAOV3 with CM-BMMSC and CAOV3 with CM-UCMSC. Second row(**h**): OVCAR3 CTR, OVCAR3 with ADMSC, OVCAR3 with BM-MSC, OVCAR3 with UC-MSC, OVCAR3 with CM-ADMSC, OVCAR3 with CM-BMMSC and OVCAR3 with CM-UCMSC. Third Row (**i**) IGROV3 CTR, IGROV3 with ADMSC, IGROV3 with BM-MSC, IGROV3 with UC-MSC, IGROV3 with CM-ADMSC, IGROV3 with CM-BMMSC and IGROV3 with CM-UCMSC. Fourth Row (**j**) SKOV3 CTR, SKOV3 with ADMSC, SKOV3 with BM-MSC, SKOV3 with UC-MSC, SKOV3 with CM-ADMSC, SKOV3 with CM-BMMSC and SKOV3 with CM-UCMSC

Cancer cells in direct co-culture with ADMSC and the CM-MSCs showed that ADMSCs, BM-MSCs, and UCMSCs induced apoptosis of SKOV3, IGROV3, CAOV3, and OVCAR3 cells. Flow cytometry was conducted to identify changes in the apoptosis rate of SKOV3, OVCAR3, IGROV3, and CAOV3 cells in co-culture with MSCs in direct contact and with the supernatant, according to Annexin V staining (Fig. 4a, b, c, d, e, f).

Our results showed cell necrosis in OVCAR3 (43.45%, *p* = 0.0119), CAOV3 (30.1%, *p* = 0.0001), SKOV3 (15.2%, p = 0.0001), and IGROV3 (35.2%, *p* = 0.0122). Necrosis was more remarkable with the supernatant derived from UCMSC compared to the supernatant of BM-MSC and ADMSC.

Rates of apoptosis were elevated in cultures of CAOV3 with the supernatant of BM-MSC (43.28%, p = 0.0001), SKOV3 with the supernatant of ADMSC (44.5%, p = 0.0001), OVCAR3 with the supernatant of UCMSC (41.97%, *p* = 0.0001), and IGROV3 with ADMSC (20.2%, p = 0.0001). Apoptosis was more significant with the supernatant derived from ADMSC compared to the supernatant derived from BM-MSC and UCMSC. This result was confirmed by DAPI blue fluorescence staining (Fig. 4g, h, i, j, k).

### Effect of MSCs on invasion and metastasis

**Fig. 5:**
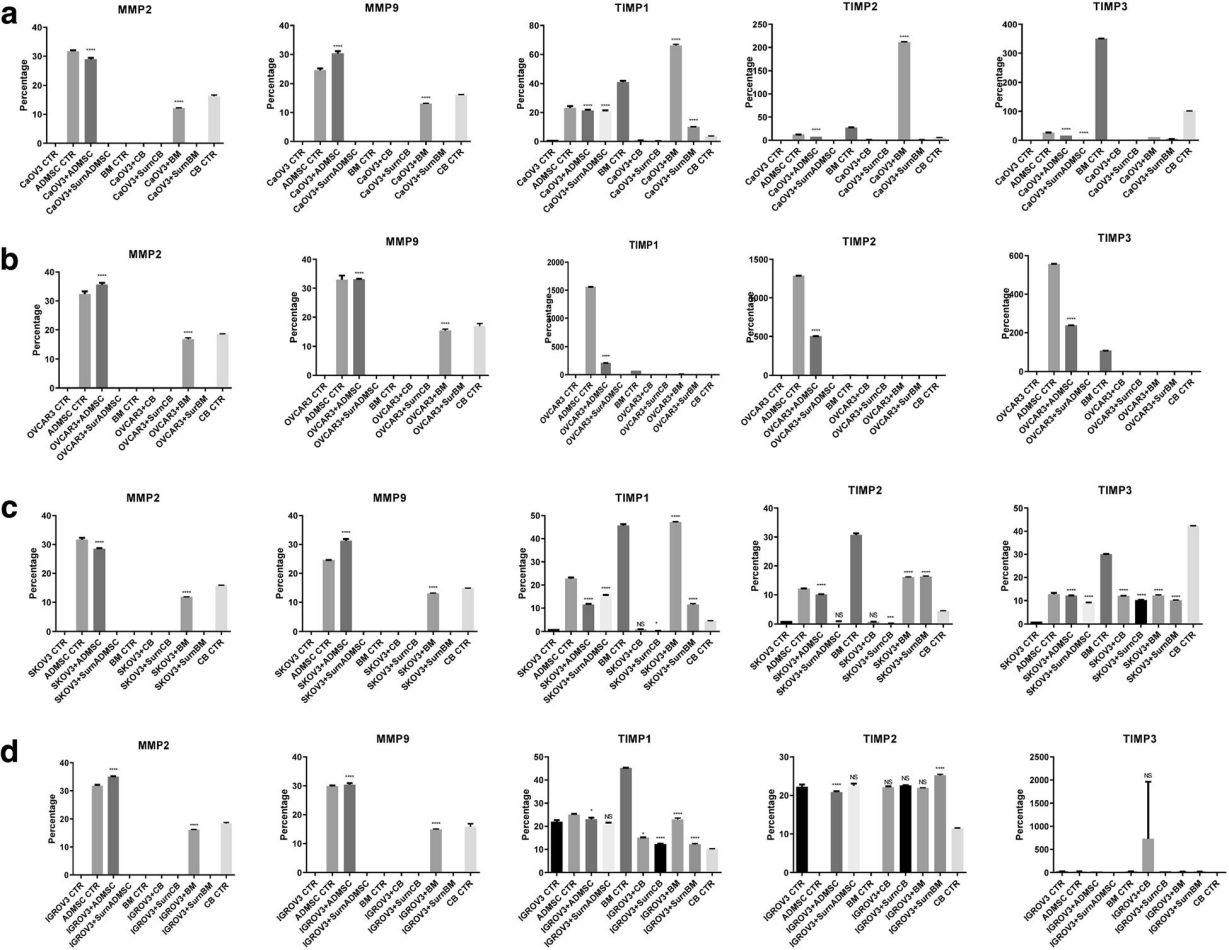
mRNA expression of MMPs and TIMPs. **a-d** Real time PCR showed a decrease in MMP-2 and MMP-9 mRNA levels, and an increase in TIMP-1, TIMP-2, and TIMP-3 mRNA levels. The results were displayed as percentage of controls, Error bars represent the means ± SD, n = 5; * p = 0.051, **p = 0.005, ***p < 0.001,****p < 0.0001

Migration and invasion are initially controlled by the dysregulated expression of MMPs and tissue inhibitor metalloproteinases (TIMPs). To examine whether TIMPs contribute to the decreased migration and invasion capacity of OVCAR3, CAOV3, IGROV3 and SKOV3 cells upon MSCs or CM-MSCs co-culture, we examined TIMP-1, − 2 and − 3 expression by real time PCR. We observed that MSCs and their CM significantly increased TIMP-1, − 2 and − 3 mRNA levels and decrease the MMP-2, MMP-9 and CA-125 mRNA expression. (Fig. 5a, b, c, d).

### Anti-inflammatory effect of MSCs

**Fig. 6:**
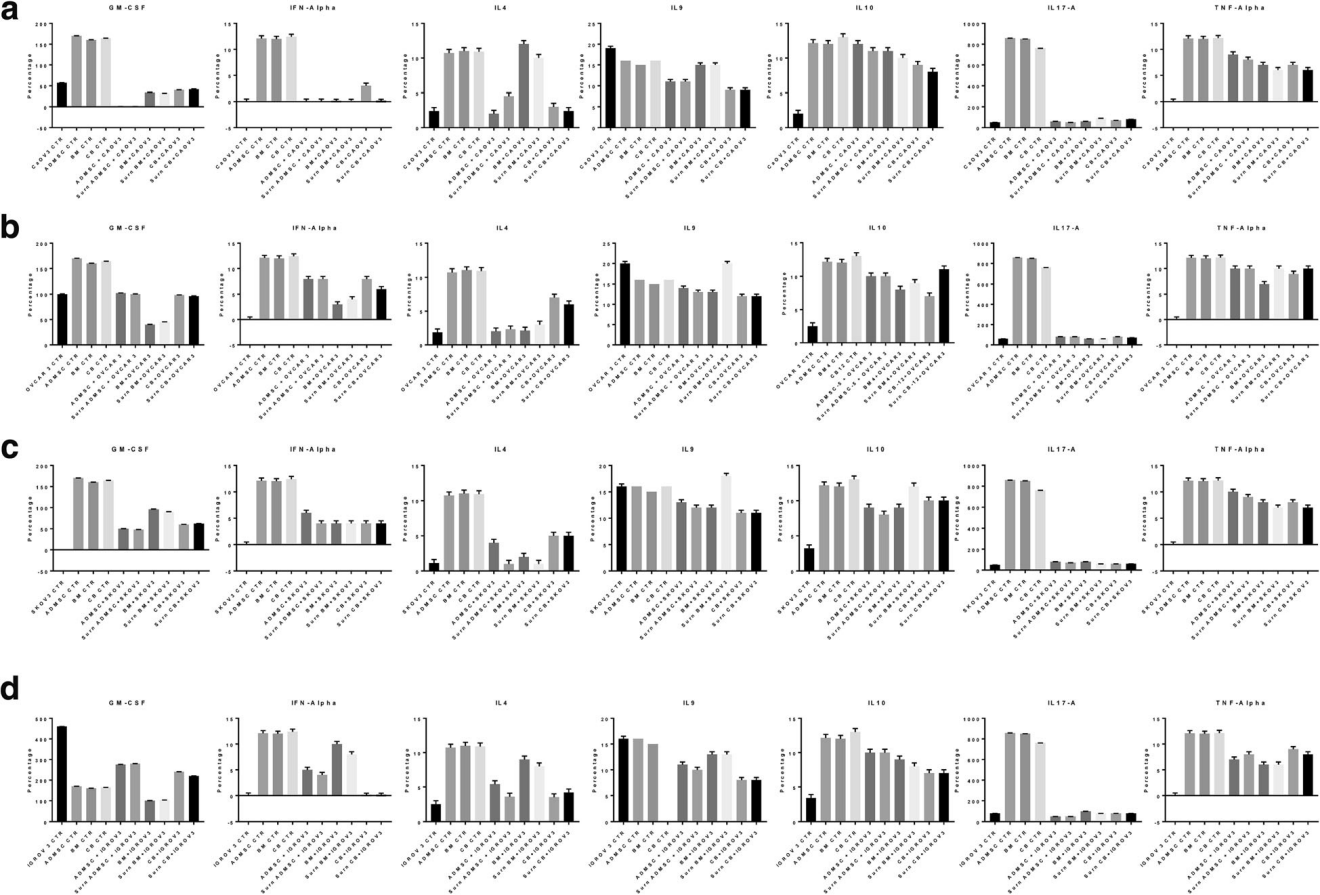
Interleukin expression. **a-d** Cell culture supernatants from co-culture were collected, and undiluted samples were analyzed for the detection of cytokines as indicated in the methods. The cytokine limits of detection (pg/ml) were: tumor necrosis factor (TNF-α) = 0.98, IFN-α = 0.99, granulocyte/macrophage colony stimulating factor (GM-CSF) = 1, and interleukins (IL-4 = 0.99, IL-9 = 0.98, IL-10 = 0.98, IL-17A = 1). The means of 5 independent experiments were performed in triplicate. *P*-value < 0.0001. Error bars represent the means ± SD, n = 5; * p = 0.051, **p = 0.005, ***p < 0.001,****p < 0.0001

The analyses of the cytokine profiles determined by flow cytometry on the collected supernatants of ADMSCs, BM-MSCs, and UCMSCs in co-culture with OVCAR3, IGROV3, CAOV3, and SKOV3 in direct contact and with the CM-MSCs showed that the levels of anti-inflammatory cytokines (IL-4, IL-10) were elevated (10–12%, *p* = 0.001) contrary to the levels of the pro-inflammatory cytokine IL-9 which decreased. Also, the levels of GM-CSF and IL-2 significantly decreased (45–50%, p = 0.001) in OVCAR3, SKOV3, and CAOV3, when co-cultured with the supernatant of ADMSCs increased in IGROV3 (25%, p = 0.0001) (Fig. 6a, b, c, d).

## Discussion

The mortality rates of ovarian cancer are increasing and no known therapy has proven efficacy in inhibiting or delaying the progression of the disease. Recently MSC has emerged as a potential therapeutic cell therapy for many diseases including some form of cancer [32, 33]. Many controversies have been reported concerning the use of MSCs in cancer, few reporting the inhibition of the tumor invasiveness and metastasis [34, 35] and others reporting an increase in tumorogenecity [36, 37]. In our paper we have proven that MSCs derived from AD, BM and UC have anti-proliferative effects and antiapoptotic on ovarian cancer. In fact, to our knowledge, this is the first in vitro study to explore the role of MSCs derived from various sources (ADMSC, BM and UC on different ovarian cell lines (OVCAR3, CAOV3, IGROV3 and SKOV3). After coculturing our cell lines with MSCs alone or with the MSC conditioned media (CM), the cancer markers CA-125, LDH, beta-HCG decreased significantly, accompanied by a decrease in the proliferation of the four cell lines. Interestingly the CD24 depicting the aggressiveness of CAOV3 and SKOV3 was inhibited more than 60%, this decrease in invasiveness was confirmed by a drastic decrease in MMP-2 and MMP-9 and increase in TIMP-1, 2 and 3 thus confirming the anti-tumorigenic effect of MSCs.

Our results are consistent with many reports suggesting that co-culture of UCMSCs and tumor cells in vitro had roles of apoptosis induction and proliferation inhibition of tumor cell [33, 37]. For example, UCMSCs displayed the obvious inhibition effects on tumorous growth in the research of malignant tumors including, melanoma, colon carcinoma, hepatocarcinoma, mammary cancer, hematological malignancy and pulmonary carcinoma [37–40] Xiufeng Li et al., showed that, with the prolonging of co-culture time of Caov-3 and hUCMSCs, the number of apoptotic CAOV3 cells became more and more; and their proliferation was inhibited remarkably where stem cell grew, ovarian cancer cells were enclosed first, then gradually necrosed and developed into clusters of granules [33]. This confirm that MSCs can alter the metastatic profile of ovarian cancer [33, 41].

CD24 is the most expressed marker in malignant hematological pathologies and several solid tumors [42–44]. Its expression correlates with cancer aggressiveness, differentiation, invasion, migration, tumorigenicity, and resistance in vitro [42]. We found by flow cytometry analysis that CD24 was significantly decreased when cocultured with MSCs and MSC-CM however the results was more significant with CM of MSCs, this is consistent with the latest theory stating that the therapeutic effects of MSCs are due to their paracrine effects and not due to their differentiation capacity [45–47]. In effect, in the report of Caplan 2019 [48] he suggested changing the name of Stem cell to medicinal signaling cells. The array of secretion or secretome have multiple therapeutic effects such as anti-inflammatory, anti-proliferative and anti-fibrotic effect beside many other roles [49–51]. The effects of the secretome has shown as anti-proliferative by many investigators such as Kalamegan et al., where they showed that Human Wharton’s Jelly Stem Cell (hWJSC) extracts inhibit ovarian cancer cell lines OVCAR3 and SKOV3 in vitro by inducing cell cycle arrest and apoptosis [52], in another study UCMSCs secretome have showed to significantly inhibit the proliferation of Skov-3 cells in addition the survival rates of ovarian cancer cells decreased with the exposure time of UCMSCs culture supernatant ie secretome [31, 33].

One of the factor secreted by MSCs are TIMPs known to play a major role in cell death and in the inhibition of the progression and metastasis of numerous cancer including Ovarian cancer [53, 54] . Inhibiting metalloproteinase might be one of the possible ways to prove the ability of MSCs to decrease and fight the aggressiveness of this cancer [55, 56]. We report in our study that the MSC-CM decreased TIMPs-1, 2, and 3, while increasing MMP-2, MMP-9. This confirm the assumption that MSCs secretome play a crucial role in cell death while inhibiting cancer progression. The decrease in MMPs and upregulation of TIMPs were also confirmed by various research groups, while others mention either no effect or a decrease in these parameters [57–59].

There are many reports declaring the link between cancer and inflammation emphasizing that chronic inflammation contributes to tumor initiation and progression [60, 61].

The anti-inflammatory cytokines play substantial role in cancer [62]. Controversial results have been reported where IL-4 and IL-10 either support or hinder tumor progression [63, 64]. In fact, it has been indicated that a lack of Il-10 allows induction of pro-inflammatory cytokines hampering anti-tumor immunity [65], and that an increased level might be used as diagnostic biomarker in certain cancer such as stomach adenocarcinoma [66]. Tanikawa et al.; have shown that a lack of IL-10 promotes tumor development, growth and metastases [63]. This was explained by the ability of Il-10 to inhibit inflammatory cytokines and the development of Treg cells and myeloid derived suppressor cells [65]. It was also that disruption from the interaction between IL-10 and it the receptors that may lead to enhanced inflammation which could promote tumor growth [63]. Il-4 was also shown to suppress cancer-directed-immunosurveillance and enhance tumor metastasis [38, 67, 68], while over-expression of IL-4 suppressed tumor development, tumor volume and weight in mice melanoma models [69]. Based on our study results, we support the anti-inflammatory effects of the cytokines and we think that by inhibiting the secretion of proinflammatory cytokines IL-10 and IL-4 will contribute to tumor suppression. We think that the paradoxal roles of the above mentioned cytokines may depend on the expressing cells as well as the molecular environments [70]. We have shown that MSCs and MSCs CM induced a significant increase in the level of IL-4 AND IL-10 in the 4 ovarian cancer cell lines.

Finally, our results suggest that MSCs and CM-MSCs inhibit tumor progression in the four different ovarian cancer cell lines. We think it is the toxic, pro-apoptotic effect of the secretion of stem cells full of proteins and growth factors with known and unknown anti-inflammatory and apoptotic activities that may contribute to the antitumor outcome [71]. Further studies are also needed to elucidate the underlying mechanism of anti-cancerous activity. A possible theory could be that the secreted cytokines or the cell to cell contact between MSCs and cancer cells stimulate the factors responsible for tumor reversions such as translationally controlled tumor protein TCTP and push the cells to acquire the knowledge on how to escape malignancy [72, 73].

## Conclusion

In conclusion, mesenchymal stem cells derived from different sources and their secretions or conditioned media appear to have a major role on inhibition of cancer aggressiveness. Although many controversies have been reported on the role of stem cells in cancers, and how crucial is that researchers continue to examine the roles and mechanisms of MSCs in tumor progression to evaluate the therapeutic potential of MSCs and to control cancer progression; the stem cells secretions arise as a new research pathway to investigate as a potential therapeutic tools for many diseases including tumor driven.

## Data Availability

The datasets used and analyzed during the current study are available from the corresponding author on reasonable request.

## References

[1] Statistics [Internet]. Ovarian Cancer Research Fund Alliance. [cited 2018 May 8]. Available from: https://ocrfa.org/patients/about-ovarian-cancer/statistics/

[2] CD44+/CD24− ovarian cancer cells demonstrate cancer stem cell properties and correlate to survival | SpringerLink [Internet]. [cited 2018 Feb 5]. Available from: https://link.springer.com/article/10.1007/s10585-012-9482-4

[3] Statistics [Internet]. Ovarian Cancer Research Fund Alliance. [cited 2018 Feb 11]. Available from: https://ocrfa.org/patients/about-ovarian-cancer/statistics/

[4] European cancer mortality predictions for the year 2018 with focus on colorectal cancer | Annals of Oncology | Oxford Academic [Internet]. [cited 2019 Jan 27]. Available from: https://academic.oup.com/annonc/article/29/4/1016/4935197 10.1093/annonc/mdy033 29562308

[5] Schellenberg A, Mauen S, Koch CM, Jans R, de Waele P, Wagner W (2014). Proof of principle: quality control of therapeutic cell preparations using senescence-associated DNA-methylation changes. BMC Research Notes.

[6] Mckinnon B, Mueller MD, Nirgianakis K, Bersinger NA (2015). Comparison of ovarian cancer markers in endometriosis favours HE4 over CA125. Mol Med Rep.

[7] Homesley HD, Filiaci V, Markman M, Bitterman P, Eaton L, Kilgore LC (2007). Phase III trial of ifosfamide with or without paclitaxel in advanced uterine carcinosarcoma: a gynecologic oncology group study. J Clin Oncol.

[8] Powell MA, Filiaci VL, Rose PG, Mannel RS, Hanjani P, Degeest K (2010). Phase II evaluation of paclitaxel and carboplatin in the treatment of carcinosarcoma of the uterus: a gynecologic oncology group study. J Clin Oncol.

[9] Penson RT, Moore KN, Herzog TJ, Burger RA, Freedman LS, Lowenton-Spier N (2018). Clinical trial in progress: A study of VB-111 combined with paclitaxel vs. paclitaxel for treatment of recurrent platinum-resistant ovarian cancer (OVAL, VB-111-701/GOG-3018). JCO.

[10] Han ES, Wen W, Dellinger TH, Wu J, Lu SA, Jove R (2018). Ruxolitinib synergistically enhances the anti-tumor activity of paclitaxel in human ovarian cancer. Oncotarget..

[11] Carboplatin/Paclitaxel Induction in Ovarian Cancer: The Finer Points | Cancer Network [Internet]. [cited 2018 Nov 5]. Available from: http://www.cancernetwork.com/ovarian-cancer/carboplatinpaclitaxel-induction-ovarian-cancer-finer-points 30153322

[12] PDQ Adult Treatment Editorial Board. Endometrial Cancer Treatment (PDQ®): Health Professional Version. In: PDQ Cancer Information Summaries [Internet]. Bethesda (MD): National Cancer Institute (US); 2002 [cited 2019 Jan 27]. Available from: http://www.ncbi.nlm.nih.gov/books/NBK65829/

[13] Uterine sarcomas - Mbatani - 2018 - International Journal of Gynecology &amp; Obstetrics - Wiley Online Library [Internet]. [cited 2019 Jan 27]. Available from: https://obgyn.onlinelibrary.wiley.com/doi/full/10.1002/ijgo.12613

[14] Rauh-Hain JA, Diver EJ, Clemmer JT, Bradford LS, Clark RM, Growdon WB (2013). Carcinosarcoma of the ovary compared to papillary serous ovarian carcinoma: a SEER analysis. Gynecol Oncol.

[15] Brown E, Stewart M, Rye T, Al-Nafussi A, Williams ARW, Bradburn M (2004). Carcinosarcoma of the ovary: 19 years of prospective data from a single center. Cancer..

[16] Mano MS, Rosa DD, Azambuja E, Ismael G, Braga S, D’Hondt V (2007). Current management of ovarian carcinosarcoma. Int J Gynecol Cancer.

[17] Ledermann JA (2018). First-line treatment of ovarian cancer: questions and controversies to address. Ther Adv Med Oncol.

[18] Lindemann K, Gao B, Mapagu C, Fereday S, Emmanuel C, Alsop K (2018). Response rates to second-line platinum-based therapy in ovarian cancer patients challenge the clinical definition of platinum resistance. Gynecol Oncol.

[19] Pittenger MF, Mackay AM, Beck SC, Jaiswal RK, Douglas R, Mosca JD (1999). Multilineage potential of adult human mesenchymal stem cells. Science..

[20] Gazit Zulma, Pelled Gadi, Sheyn Dmitriy, Yakubovich Doron C., Gazit Dan (2019). Mesenchymal Stem Cells. Principles of Regenerative Medicine.

[21] Kim J, Hematti P (2009). Mesenchymal stem cell-educated macrophages: a novel type of alternatively activated macrophages. Exp Hematol.

[22] Prockop DJ, Oh JY (2012). Mesenchymal stem/stromal cells (MSCs): role as guardians of inflammation. Mol Ther.

[23] Mohr A, Zwacka R (2018). The future of mesenchymal stem cell-based therapeutic approaches for cancer - from cells to ghosts. Cancer Lett.

[24] Stoma I, Karpov I, Krivenko S, Iskrov I, Milanovich N, Koritko A (2018). Mesenchymal stem cells transplantation in hematological patients with acute graft-versus-host disease: characteristics and risk factors for infectious complications. Ann Hematol.

[25] Kfoury Y, Scadden DT (2015). Mesenchymal cell contributions to the stem cell niche. Cell Stem Cell.

[26] Kern S, Eichler H, Stoeve J, Klüter H, Bieback K (2006). Comparative analysis of mesenchymal stem cells from bone marrow, umbilical cord blood, or adipose tissue. Stem Cells.

[27] Matuskova M, Durinikova E, Altaner C, Kucerova L (2018). Genetically engineered mesenchymal stromal cells in cancer gene therapy. Bratisl Lek Listy.

[28] Hass R, Kasper C, Böhm S, Jacobs R (2011). Different populations and sources of human mesenchymal stem cells (MSC): a comparison of adult and neonatal tissue-derived MSC. Cell Commun Signal.

[29] Mahmoudifar N, Doran PM (2015). Mesenchymal stem cells derived from human adipose tissue. Methods Mol Biol.

[30] Sibov TT, Severino P, Marti LC, Pavon LF, Oliveira DM, Tobo PR (2012). Mesenchymal stem cells from umbilical cord blood: parameters for isolation, characterization and adipogenic differentiation. Cytotechnology..

[31] Shi Q, Gao J, Jiang Y, Sun B, Lu W, Su M, et al. Differentiation of human umbilical cord Wharton’s jelly-derived mesenchymal stem cells into endometrial cells. Stem Cell Res Ther [Internet]. 2017:8 Available from: https://www.ncbi.nlm.nih.gov/pmc/articles/PMC5667478/. 10.1186/s13287-017-0700-5 PMC5667478 29096715

[32] Zhou Y-L, Li Y-M, He W-T (2019). Oxygen-laden mesenchymal stem cells enhance the effect of gastric cancer chemotherapy in vitro. Oncol Lett.

[33] Li X, Li Z (2019). Effects of human umbilical cord mesenchymal stem cells on co-cultured ovarian carcinoma cells. Microsc Res Tech.

[34] Serhal R, Saliba N, Hilal G, Moussa M, Hassan GS, El Atat O (2019). Effect of adipose-derived mesenchymal stem cells on hepatocellular carcinoma: in vitro inhibition of carcinogenesis. World J Gastroenterol.

[35] Gao D, Mittal V, Ban Y, Lourenco AR, Yomtoubian S, Lee S (2018). Metastatic tumor cells - genotypes and phenotypes. Front Biol (Beijing).

[36] Melzer Catharina, von der Ohe Juliane, Hass Ralf (2019). In Vivo Cell Fusion between Mesenchymal Stroma/Stem-Like Cells and Breast Cancer Cells. Cancers.

[37] Zhou J, Tan X, Tan Y, Li Q, Ma J, Wang G (2018). Mesenchymal stem cell derived exosomes in Cancer progression, metastasis and drug delivery: a comprehensive review. J Cancer.

[38] Liu Y-J, Dou X-Q, Wang F, Zhang J, Wang X-L, Xu G-L (2017). IL-4Rα aptamer-liposome-CpG oligodeoxynucleotides suppress tumour growth by targeting the tumour microenvironment. J Drug Target.

[39] Alshareeda AT, Rakha E, Alghwainem A, Alrfaei B, Alsowayan B, Albugami A (2018). The effect of human placental chorionic villi derived mesenchymal stem cell on triple-negative breast cancer hallmarks. PLoS One.

[40] Ling X, Marini F, Konopleva M, Schober W, Shi Y, Burks J (2010). Mesenchymal stem cells overexpressing IFN-β inhibit breast Cancer growth and metastases through Stat3 signaling in a syngeneic tumor model. Cancer Microenviron.

[41] Mooney R, Majid AA, Batalla-Covello J, Machado D, Liu X, Gonzaga J (2019). Enhanced delivery of oncolytic adenovirus by neural stem cells for treatment of metastatic ovarian Cancer. Mol Ther Oncolytics.

[42] Kristiansen G, Denkert C, Schlüns K, Dahl E, Pilarsky C, Hauptmann S (2002). CD24 is expressed in ovarian Cancer and is a new independent prognostic marker of patient survival. Am J Pathol.

[43] Tarhriz V, Bandehpour M, Dastmalchi S, Ouladsahebmadarek E, Zarredar H, Eyvazi S (2019). Overview of CD24 as a new molecular marker in ovarian cancer. J Cell Physiol.

[44] Tao Y, Li H, Huang R, Mo D, Zeng T, Fang M (2018). Clinicopathological and prognostic significance of Cancer stem cell markers in ovarian Cancer patients: evidence from 52 studies. CPB..

[45] Mancuso P, Raman S, Glynn A, Barry F, Murphy JM (2019). Mesenchymal stem cell therapy for osteoarthritis: the critical role of the cell Secretome. Front Bioeng Biotechnol.

[46] Sriramulu S, Banerjee A, Di Liddo R, Jothimani G, Gopinath M, Murugesan R (2018). Concise review on clinical applications of conditioned medium derived from human umbilical cord-mesenchymal stem cells (UC-MSCs). Int J Hematol Oncol Stem Cell Res.

[47] Gunawardena TNA, Mohammad TR, Abdullah BJJ, Abu Kasim NH (2019). Conditioned media serived from mesenchymal stem cell cultures: the next generation for regenerative medicine. J Tissue Eng Regen Med.

[48] Caplan AI (2019). Medicinal signalling cells: they work, so use them. Nature..

[49] Sutton MT, Fletcher D, Episalla N, Auster L, Kaur S, Gwin MC, et al. Mesenchymal Stem Cell Soluble Mediators and Cystic Fibrosis. J Stem Cell Res Ther. 2017;7(9). 10.4172/2157-7633.1000400 PMC5747256 29291140

[50] Jalili Angourani K, Mazhari S, Farivar S, Salman Mahini D, Rouintan A, Baghaei K (2018). Fibroblast-myofibroblast crosstalk after exposure to mesenchymal stem cells secretome. Gastroenterol Hepatol Bed Bench.

[51] Fernandes-Cunha GM, Na K-S, Putra I, Lee HJ, Hull S, Cheng Y-C, et al. Corneal Wound Healing Effects of Mesenchymal Stem Cell Secretome Delivered Within a Viscoelastic Gel Carrier. Stem Cells Transl Med. 2019. 10.1002/sctm.18-0178 PMC6477005 30644653

[52] Kalamegam G, Sait KHW, Ahmed F, Kadam R, Pushparaj PN, Anfinan N, et al. Human Wharton’s Jelly Stem Cell (hWJSC) Extracts Inhibit Ovarian Cancer Cell Lines OVCAR3 and SKOV3 in vitro by Inducing Cell Cycle Arrest and Apoptosis. Front Oncol [Internet]. 2018;8. [cited 2019 Mar 5]. Available from: https://www.frontiersin.org/articles/10.3389/fonc.2018.00592/full 10.3389/fonc.2018.00592 PMC6293270 30581772

[53] Będkowska GE, Gacuta E, Zajkowska M, Głażewska EK, Osada J, Szmitkowski M (2017). Plasma levels of MMP-7 and TIMP-1 in laboratory diagnostics and differentiation of selected histological types of epithelial ovarian cancers. J Ovarian Res.

[54] Zhang Y, Chen Q (2017). Relationship between matrix metalloproteinases and the occurrence and development of ovarian cancer. Braz J Med Biol Res.

[55] Almalki SG, Agrawal DK. Effects of matrix metalloproteinases on the fate of mesenchymal stem cells. Stem Cell Res Ther [Internet]. 2016;7(1). [cited 2019 Jul 8]. Available from: https://www.ncbi.nlm.nih.gov/pmc/articles/PMC5016871/10.1186/s13287-016-0393-1 PMC5016871 27612636

[56] Ries C, Egea V, Karow M, Kolb H, Jochum M, Neth P (2007). MMP-2, MT1-MMP, and TIMP-2 are essential for the invasive capacity of human mesenchymal stem cells: differential regulation by inflammatory cytokines. Blood..

[57] Hu X, Li D, Zhang W, Zhou J, Tang B, Li L (2012). Matrix metalloproteinase-9 expression correlates with prognosis and involved in ovarian cancer cell invasion. Arch Gynecol Obstet.

[58] Boyd RS, Balkwill FR (1999). MMP-2 release and activation in ovarian carcinoma: the role of fibroblasts. Br J Cancer.

[59] Al-Alem L, Curry TE (2015). Ovarian cancer: involvement of the matrix metalloproteinases. Reproduction..

[60] DiDonato JA, Mercurio F, Karin M (2012). NF-κB and the link between inflammation and cancer. Immunol Rev.

[61] Ahechu P, Zozaya G, Martí P, Hernández-Lizoáin JL, Baixauli J, Unamuno X (2018). NLRP3 Inflammasome: a possible link between obesity-associated low-grade chronic inflammation and colorectal Cancer development. Front Immunol.

[62] Li M, Kouzmina E, McCusker M, Rodin D, Boutros PC, Paige CJ (2017). Pro- and anti-inflammatory cytokine associations with major depression in cancer patients. Psychooncology..

[63] Tanikawa T, Wilke CM, Kryczek I, Chen GY, Kao J, Núñez G (2012). Interleukin (IL)-10 ablation promotes tumor development, growth and metastasis. Cancer Res.

[64] Mocellin S, Panelli MC, Wang E, Nagorsen D, Marincola FM (2003). The dual role of IL-10. Trends Immunol.

[65] Acuner-Ozbabacan ES, Engin BH, Guven-Maiorov E, Kuzu G, Muratcioglu S, Baspinar A (2014). The structural network of Interleukin-10 and its implications in inflammation and cancer. BMC Genomics.

[66] Shokrzadeh M, Mohammadpour A, Hoseini V, Abediankenari S, Ghassemi-Barghi N, Tabari YS (2018). Serum cytokine of IL-2, IL-10 and IL-12 levels in patients with stomach adenocarcinoma. Arq Gastroenterol.

[67] Rezaeishahmirzadi M, Motamedi Rad N, Kalantar M, Ayatollahi H, Shakeri S, Sheikhi M (2018). The Association of Gastritis and Peptic Ulcer with Polymorphisms in the inflammatory-related genes IL-4 and IL-10 in Iranian population. Iran J Pathol.

[68] Guruprasath P, Kim J, Gunassekaran GR, Chi L, Kim S, Park R-W (2017). Interleukin-4 receptor-targeted delivery of Bcl-xL siRNA sensitizes tumors to chemotherapy and inhibits tumor growth. Biomaterials..

[69] Lee HL, Park MH, Song JK, Jung YY, Kim Y, Kim KB (2016). Tumor growth suppressive effect of IL-4 through p21-mediated activation of STAT6 in IL-4Rα overexpressed melanoma models. Oncotarget..

[70] Li Z, Chen L, Qin Z (2009). Paradoxical roles of IL-4 in tumor immunity. Cell Mol Immunol.

[71] Proietti S, Cucina A, Pensotti A, Biava PM, Minini M, Monti N (2019). Active fraction from embryo fish extracts induces reversion of the malignant invasive phenotype in breast Cancer through Down-regulation of TCTP and modulation of E-cadherin/β-catenin pathway. Int J Mol Sci.

[72] Tuynder M, Fiucci G, Prieur S, Lespagnol A, Géant A, Beaucourt S (2004). Translationally controlled tumor protein is a target of tumor reversion. Proc Natl Acad Sci U S A.

[73] Amson R, Karp JE, Telerman A (2013). Lessons from tumor reversion for cancer treatment. Curr Opin Oncol.

